# Behavioral Immune Trade-Offs: Interpersonal Value Relaxes Social Pathogen Avoidance

**DOI:** 10.1177/0956797620960011

**Published:** 2020-09-17

**Authors:** Joshua M. Tybur, Debra Lieberman, Lei Fan, Tom R. Kupfer, Reinout E. de Vries

**Affiliations:** 1 Department of Experimental and Applied Psychology, Vrije Universiteit Amsterdam; 2 Institute of Brain and Behavior Amsterdam; 3 Department of Psychology, University of Miami

**Keywords:** infectious disease, behavioral immune system, disgust, evolutionary psychology, welfare trade-offs, open data, open materials, preregistered

## Abstract

Behavioral-immune-system research has illuminated how people detect and avoid signs of infectious disease. But how do we regulate exposure to pathogens that produce no symptoms in their hosts? This research tested the proposition that estimates of interpersonal value are used for this task. The results of three studies (*N* = 1,694), each conducted using U.S. samples, are consistent with this proposition: People are less averse to engaging in infection-risky acts not only with friends relative to foes but also with honest and agreeable strangers relative to dishonest and disagreeable ones. Further, a continuous measure of how much a person values a target covaries with comfort with infection-risky acts with that target, even within relationship categories. Findings indicate that social prophylactic motivations arise not only from cues to infectiousness but also from interpersonal value. Consequently, pathogen transmission within social networks might be exacerbated by relaxed contamination aversions with highly valued social partners.

Everyday life involves navigating a minefield of infectious microbes that aim to exploit our bodies for their own gain. We deftly avoid most of these pathogens, as if we have some awareness of where they lie, despite their invisibility to the naked eye. Such avoidance is often affectively motivated. For example, the scents of bodily wastes—reliable sources of pathogens throughout our evolutionary history—elicit disgust, which motivates contact avoidance (Tybur, Lieberman, Kurzban, & DeScioli, 2013). Similar avoidance occurs socially; people shun those unlucky enough to display many infectious disease symptoms, including the pustules caused by smallpox, the asymmetric swellings caused by mumps, and the fluid-filled lesions caused by yaws (Oaten, Stevenson, & Case, 2011). Understanding responses to these and other cues to pathogens has formed the bedrock of behavioral-immune-system research (Ackerman, Hill, & Murray, 2018; Murray & Schaller, 2016; Neuberg, Kenrick, & Schaller, 2011).

Yet a behavioral immune system that motivates avoidance of only individuals covered in rashes, pox, or swellings would leave us exposed to myriad pathogens transmitted by individuals showing no signs of illness. Consider the consequences of contact with the early 20th century cook Mary Mallon (“Typhoid Mary”), who transmitted sometimes-lethal typhoid infections to dozens of people despite showing no symptoms of illness herself. Similar asymptomatic transmission is common across infectious agents. For example, volunteer infection studies indicate that 90% of participants dosed with influenza shed viral particles, but only 70% show symptoms (Carrat et al., 2008). Among people who do eventually become ill, viral shedding begins before symptoms appear and peaks before illness does. Asymptomatic transmission is typical of many sexually transmitted infections (Farley, Cohen, & Elkins, 2003), and it appears to underlie much of the spread of the SARS-CoV-2 virus that causes COVID-19 (Li et al., 2020). Further, a person can transmit pathogens without being infected, simply by touching a pathogen-contaminated surface. Ultimately, every person can transmit pathogens, and apparent health tells little about many common infection threats.

How do people navigate a social world in which infectious agents are ubiquitous yet often undetectable, even to a behavioral immune system that seems tailored to detecting and neutralizing pathogens?

## Trade-Offs: The Costs and Benefits of Mitigating Exposure to Pathogens

As highlighted in the behavioral-immune-system literature, investments in pathogen avoidance often impose costs on other fitness-promoting behaviors (e.g., Oaten, Stevenson, & Case, 2009; Schaller, 2015; Tybur & Lieberman, 2016). Consider the most severe and most relaxed pathogen-avoidance strategies possible. On one extreme, we could experience motivations to avoid all direct and indirect contact with all people. While minimizing exposure to pathogens, such motivations would largely eliminate food sharing, sexual behavior, cooperation on joint tasks, and aid to kin and romantic partners (e.g., Case, Repacholi, & Stevenson, 2006; Fleischman, Hamilton, Fessler, & Meston, 2015). On the other extreme, we could experience no motivations to avoid direct and indirect contact—we could feel comfortable touching or licking any person or any object touched by another person. This approach, while eliminating the social costs of contact avoidance, would leave us severely vulnerable to infection. A well-designed behavioral immune system should instead balance the costs of pathogen exposure against those of social avoidance in a target-specific manner. Guided by the considerations described above, researchers have uncovered evidence that mandrills groom parasitized maternal kin but avoid grooming other parasitized conspecifics (Poirotte & Charpentier, 2020) and that human mothers report less disgust toward their own baby’s diapers than other babies’ diapers (Case et al., 2006).

**Statement of Relevance**

People deftly navigate around pathogens, including those hiding in bodily wastes, spoiled foods, and individuals with infectious disease symptoms, even without consciously considering the consequences of infection. They do so because natural selection has shaped our sensory and motivational systems as a kind of behavioral immune system. However, many pathogen threats, including those posed by asymptomatic influenza and COVID-19 carriers, show no signs of infectiousness. The current work uncovers new information regarding how people navigate these types of infection threats. Results from three studies indicate that people feel strongly motivated to avoid infection-risky behaviors with unsavory strangers and disliked acquaintances, but they are more comfortable taking identical risks with individuals whose welfare they value. These findings may help explain epidemiological patterns such as family-group clustering: Infections spread not only because of proximity but also because of greater comfort with exposure to the unseen pathogens transmitted by people we value.

More broadly, disgust “source effects” are consistent with the idea that similar trade-offs operate outside the kinship domain; for example, some studies have found that people imagine the bodily fluids or wastes from a friend to be less aversive than those from a stranger (Curtis, Aunger, & Rabie, 2004; Peng, Chang, & Zhou, 2013; Rozin, Nemeroff, Wane, & Sherrod, 1989; Stevenson & Repacholi, 2005). Rather than reflecting lower pathogen avoidance toward more valued conspecifics, though, these findings have been interpreted as suggesting that familiarity is treated as information regarding infection threat, just as pustules and lesions are. In the current study, we tested the alternative account described above: that willingness to engage in infection-risky behaviors tracks interpersonal value, even in the absence of illness symptoms.

## Interpersonal Value Between and Within Categories

Interpersonal value does not map neatly onto categories labeled with terms such as family, friend, and foe. The category “kin” alone reflects multiple relationship types (e.g., parent, offspring, sibling, half-sibling), and relationships are differentially valued within such categories (e.g., siblings; Sznycer, De Smet, Billingsley, & Lieberman, 2016). Strangers also vary in interpersonal affordances: Some are more likely to become valuable exchange partners, and others are more likely to inflict social costs. Hence, if social pathogen avoidance tracks perceptions of interpersonal value, then people should be more comfortable with infection-risky behaviors not only with individuals from less valued categories but also with more interpersonally valued targets within categories.

Although interpersonal value is strongly influenced by kinship, it is also shaped by, among other things, mutual valuation, as occurs in friendships, and inclinations to engage in reciprocity, as occurs in exchange partners (Tooby & Cosmides, 1996). These disparate sources of benefits are putatively integrated into a welfare-trade-off ratio (WTR)—an individual’s willingness to trade off his or her welfare for that of another (Delton & Robertson, 2016; Kirkpatrick, Delton, Robertson, & de Wit, 2015; Smith, Pedersen, Forster, McCullough, & Lieberman, 2017; Tooby, Cosmides, Sell, Lieberman, & Sznycer, 2008). We used tasks that measure willingness to trade-off one’s own welfare for that of another to investigate whether comfort with potentially infectious contact tracks interpersonal value.

## Overview of the Present Studies

Across three studies, we tested the hypothesis that motivations to avoid infection-risky behaviors relate to target-specific interpersonal value. In Studies 1 and 2, participants reported their comfort with infection-risky acts with a target they know personally (either a romantic partner, a friend, an acquaintance, or a disliked other), and they completed a target-specific WTR task. In Study 3, participants reported their comfort with these same infection-risky acts with a stranger, who was described as either high or low on honesty-humility and agreeableness, two personality traits that should inform expected interpersonal value. Participants from each study were U.S. residents recruited using Amazon’s Mechanical Turk. Samples drawn from this pool are similar to nationally representative samples in many ways, though they tend to be a bit younger, less religious, and less politically conservative (Levay, Freese, & Druckman, 2016). Preregistrations, data, and R analysis scripts for all three studies are available on OSF (https://osf.io/4agk8/).

## Study 1

### Method

Study 1 examined whether people are less avoidant of potentially infectious contact with individuals from more valuable relationship categories and whether interpersonal value predicts pathogen avoidance within categories.

#### Participants

We preregistered a target of 500 participants. We did not use an a priori effect-size estimate, though this sample afforded 80% power to detect a small effect size (*r*) of .12. Five hundred four individuals (55.16% male; age: *M* = 35.88 years, *SD* = 10.2) participated in exchange for $1.50. All respondents provided informed consent.

#### Procedure

After reporting demographic information (e.g., sex, age, relationship status, income), each participant was randomly assigned to think of either (a) their romantic partner, (b) their closest friend, (c) an acquaintance, or (d) someone they know personally but dislike. Participants who had previously reported being in a romantic relationship had a 40% chance of being assigned to the romantic-partner condition and a 20% chance of being assigned to each of the other three conditions; single participants had a 33% chance of being assigned to the three non-romantic-partner conditions. Participants were first asked to write the target’s initials, which appeared in the remaining questions about the target. They were then asked to write a few sentences describing the target’s physical appearance, to report how long they have known the target, and to report the target’s age and sex.

To measure motivations to avoid pathogen exposure, we generated 10 items inspired by the germ-aversion subscale from the Perceived Vulnerability to Disease scale (Duncan, Schaller, & Park, 2009). Example items included “Using [target]’s deodorant stick on yourself,” “Wearing a hat that [target] has worn many times,” and “Touching a handkerchief that [target] used to blow his or her nose.” Participants rated each item on a scale from −3 (*very uncomfortable*) to 3 (*very comfortable*), with the midpoint labeled 0 (*neutral*). A principal-axis factor analysis suggested that these items varied along a single dimension (all factor loadings were above 0.74; α = .96). Mean contact comfort was 0.06 (*SD* = 1.97). Lower scores were interpreted as corresponding with greater motivations to avoid exposure to the pathogens potentially transmitted by the target.

To assess interpersonal value, we used a WTR task (Delton & Robertson, 2016; Kirkpatrick et al., 2015; Smith et al., 2017). In this task, participants are asked to select one of two options, the first of which involves the participant receiving money, and the second of which involves the target receiving money. Each target-benefiting decision is characterized by a different welfare trade-off—that is, a different ratio of benefits received by the target relative to what could have been received by the participant. For example, for one of the items, participants decided whether they would rather receive $17 with the target receiving nothing or receive nothing with the target receiving $37. Choosing the beneficial option for the target would imply a WTR toward that target of at least 0.45 (i.e., 17/37). Participants completed the same 60 items described by Kirkpatrick et al. (2015), which include six anchor points (fixed values received by the target), each of which has 10 values that the participant would receive. Switch points—the ratio at which participants begin choosing the benefit for the target—were calculated for each anchor and averaged (α = .99). Further details are provided in the Supplemental Material available online.

We also asked participants to rate the target’s honesty-humility (for an overview, see de Vries, Tybur, Pollet, & van Vugt, 2016) using the 10 honesty-humility items from the HEXACO-60 (Ashton & Lee, 2009; α = .88). Individuals higher in honesty-humility report less willingness to exploit others (Van Gelder & de Vries, 2012), and they behave more prosocially in tasks with financial consequences (e.g., returning more money in trust games, offering more money in dictator games; Thielmann, Spadaro, & Balliet, 2020). In sum, partners higher in honesty-humility are more likely to confer benefits in social relationships and hence should be more valued as relationship partners.

Finally, we also measured the extent to which participants felt generally motivated to avoid pathogen cues using the seven-item pathogen domain of the Three Domain Disgust Scale (Tybur, Lieberman, & Griskevicius, 2009), which asks participants to rate seven items (e.g., “Stepping in dog poop”) on a scale from 0 (*not at all disgusting*) to 6 (*extremely disgusting*; α = .83).

#### Data exclusion

We excluded participants with more than two switch points within any of the six WTR anchors (*n* = 35), three participants whose descriptions of their partners were nonsensical or demonstrated poor English, and two participants who selected a gender option indicating that they were neither a man nor a woman. These latter participants were excluded so that sex differences could be examined. Results reported below are based on the remaining 464 participants. All outcomes of null-hypothesis significance testing (i.e., *p* < .05) remained when no exclusions were made.

### Results

**Fig. 1.**
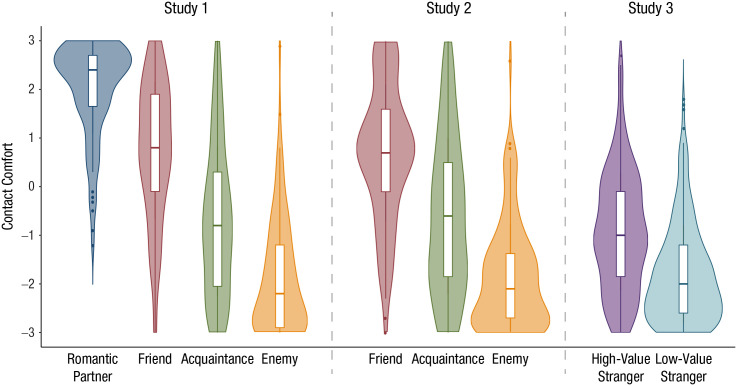
Mean comfort with infection-risky contact with each target type, separately for Studies 1 through 3. In each violin plot, the horizontal line indicates the median, the white box indicates the interquartile range of the data, the shaded area indicates the density of the data, and the whiskers extend 1.5 times the interquartile range. Outliers are indicated by dots.

**Fig. 2.**
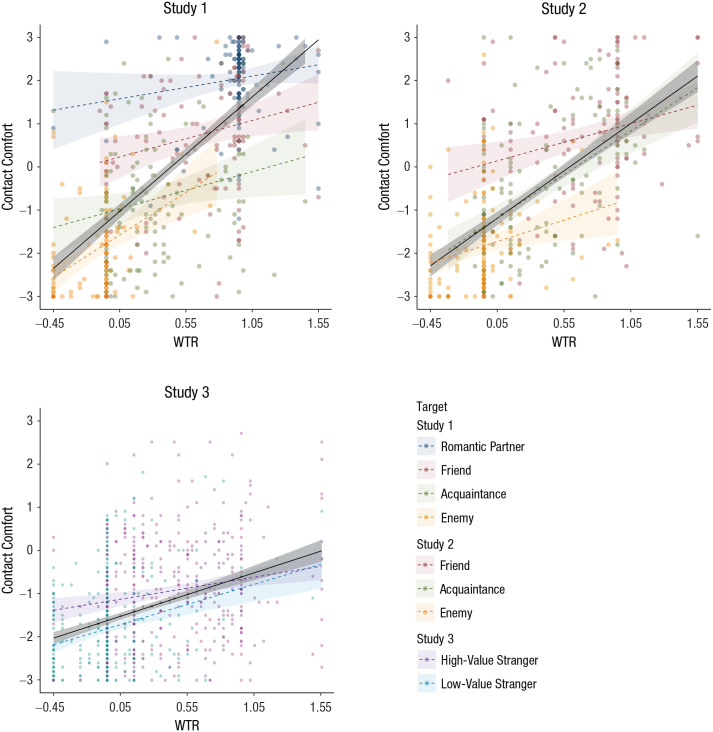
Relations between welfare-trade-off ratio (WTR) and comfort with infection-risky contact, separately for each target type in Studies 1 through 3. In each scatterplot, the solid line indicates the best-fitting relationship between WTR and contact comfort across categories, and the dashed lines indicate the best-fitting relationship within each target category. Shaded areas around the regression lines indicate 95% confidence intervals.

Participants were more comfortable with potentially infectious contact with targets whose welfare they valued, *r* = .68, 95% confidence interval (CI) = [.63, .73], *p* < .001 (see Fig. 2), and with targets rated as higher on honesty-humility, *r* = .47, 95% CI = [.40, .54], *p* < .001. A number of other variables also related to contact comfort, including sensitivity to pathogen disgust, *r* = −.22, 95% CI = [−.30, −.13], *p* < .001, and target sex, with participants reporting greater comfort with infectious contact with women than with men, *r* = .17, 95% CI = [.08, .26], *p* < .001. Notably, the main effect of target sex was qualified by an interaction with participant sex^1^ (details are provided in the Supplemental Material). Critically, contact comfort also varied across relationship type (romantic partner, close friend, acquaintance, enemy), *F*(3, 460) = 213.52, *p* < .001, η^2^ = .58, 90% CI = [.54, .62] (see Fig. 1), as did WTR and honesty-humility (for target-category differences in WTR and honesty-humility and a full correlation matrix, see the Supplemental Material).

We next conducted hierarchical regression analyses to test whether WTR value relates to contact comfort independently of relationship type. In a first step (adjusted *R*^2^ = .08; see Fig. 2), contact comfort was regressed on variables unrelated to WTR, including participant sex and income, target sex, and pathogen-disgust sensitivity. Adding WTR, *b* = 2.36, *p* < .001, *r_p_*^2^ = .33, 90% CI = [.27, .39], and target honesty-humility, *b* = 0.19, *p* = .03, *r_p_*^2^ = .03, 90% CI = [.005, .06], to the model accounted for an additional 42.82% of the variance in contact comfort. But were these effects of WTR entirely accounted for by the category of partner that participants were asked to imagine? No. Although the third step incorporating three orthogonally coded variables representing the four relationship categories accounted for an additional 13.43% of variance in contact comfort, WTR continued to account for unique variance, *b* = 0.85, *p* < .001, *r_p_*^2^ = .05, 90% CI = [.01, .08], though target honesty-humility did not, *b* = 0.05, *p* = .49, *r_p_*^2^ < .001, 90% CI = [−.01, .01].

## Study 2

### Method

Study 2 closely mirrored Study 1, with four exceptions. First, given that WTR, rather than target-rated honesty-humility, uniquely related to contact comfort, we did not assess target honesty-humility. We instead assessed participants’ prosocial personality traits, which might jointly relate to WTR and pathogen avoidance (Kirkpatrick et al., 2015; Kupfer & Tybur, 2017). Second, given asymmetries in target sex across the four categories used in Study 1 (4%, 70%, 76%, and 74% same-sex for romantic partner, closest friend, acquaintance, and disliked other, respectively), we randomly assigned each participant to picture either a male or a female target. To accommodate this change, we eliminated the romantic-partner condition.

#### Participants

We preregistered a recruitment target of 430 individuals, which we anticipated would be reduced to approximately 387 after exclusions. This sample size was targeted to facilitate exploratory analyses involving participant sex and target sex (see the Supplemental Material), and it provided more than 99% power to detect the relation between WTR and contact comfort observed in Study 1. We recruited only participants not enrolled in Study 1. Four hundred thirty individuals (56.28% male; age: *M* = 36.29 years, *SD* = 10.99) participated in exchange for $2.00. All respondents provided informed consent.

#### Procedures

Procedures were identical to those in Study 1, with a few notable exceptions. First, each participant was randomly assigned to picture either a man or a woman from one of the three categories (i.e., closest male friend, closest female friend, male acquaintance, female acquaintance, male disliked other, or female disliked other). Second, they provided self-reports of agreeableness (α = .84) and honesty-humility (α = .81; rather than target ratings of honesty-humility) from the HEXACO-60 (Ashton & Lee, 2009). Third, given high consistency across the six anchor points used in Study 1, they completed a 30-item WTR measure rather than the 60-item version (α = .97). They also completed a handful of additional items, which were not included in our preregistered analysis plan (see the Supplemental Material).

#### Data exclusion

We excluded 19 participants with more than two switch points within any of the three WTR anchors, seven participants whose descriptions of their partners were nonsensical or demonstrated poor English, and one participant who described their gender identity as neither male nor female. The results reported below are based on the remaining 403 participants. All outcomes of null-hypothesis significance testing (i.e., *p* < .05) remained when no exclusions were made.

### Results

As in Study 1, participants were more comfortable with potentially infectious contact with more interpersonally valued targets, *r* = .61, 95% CI = [.54, .67], *p* < .001 (see Fig. 2). Contact comfort also related to pathogen-disgust sensitivity, *r* = −.24, 95% CI = [−.33, −.15], *p* < .001. And, as expected, it also varied across relationship type (close friend, acquaintance, enemy), *F*(2, 397) = 116.7, *p* < .001, η^2^ = .37, 90% CI = [.31, .42] (see Fig. 1). A full list of correlations is provided in the Supplemental Material.

We next ran our preregistered analyses, in which we first entered participant characteristics (sex, age, self-reports of honesty-humility, agreeableness—as opposed to target reports used in Study 1—and pathogen-disgust sensitivity), then target characteristics (sex, age, WTR) in a second step, then relationship category in a third step. Participant characteristics accounted for approximately 6.16% of the variance in contact comfort, and target characteristics apart from relationship category accounted for an additional 36.82% of the variance. In that second step, WTR was most strongly related to contact comfort, *b* = 2.06, *p* < .001, *r_p_*^2^ = .36, 90% CI = [.29, .42]. Adding relationship category accounted for an additional 7.77% of the variance in contact comfort, but, as in Study 1, the unique effect of WTR remained, *b* = 1.13, *p* < .001, *r_p_*^2^ = .09, 90% CI = [.05, .14].

## Study 3

### Method

Study 3 was designed to address limitations of Studies 1 and 2: Each participant pictured a different target, and unmeasured third variables could have confounded pathogen-avoidance motivations and interpersonal value. For example, given that people with many symptoms of illness are socially devalued (Oaten et al., 2011), less interpersonally valued partners might actually be more infectious, and the relation between WTR and pathogen-avoidance motivations could have reflected stronger avoidance of more infectious individuals. To address this limitation, we accounted for the identity of the target in Study 3. Participants saw a picture of a stranger and read a description containing information about that person’s value as an exchange partner. Given that individuals higher in honesty-humility and agreeableness behave more prosocially in social dilemmas (e.g., dictator games, trust games, ultimatum games; Thielmann, Spadaro, & Balliet, 2020), and such games serve as abstractions of valuable behavior in exchange relationships (e.g., willingness to share resources, trust, forgiveness; Murnighan & Wang, 2016), we designed the descriptions to communicate either high honesty-humility and agreeableness or low honesty-humility and agreeableness. Similar types of information about strangers have been shown to influence the magnitude of WTRs in the expected direction (e.g., Smith et al., 2017). We predicted that motivations to avoid pathogens would be higher for targets low in honesty-humility and agreeableness, because these targets offer little benefits that might offset potential costs of infection, and that target-specific WTR would again relate to willingness to expose oneself to unseen pathogens. Notably, all targets were strangers to participants, and any differences in contact comfort across high versus low agreeableness and honesty-humility targets cannot be explained by putatively greater infection threats posted by strangers relative to friends (e.g., Peng et al., 2013).

#### Participants

We preregistered a recruitment target of 870 individuals, which we anticipated would be reduced to approximately 800 after exclusions. This sample size afforded 80% power assuming a small effect of the manipulation (*d* = 0.20), with a target intercept variance component of .15 and a target slope variance of .02 (Westfall et al., 2014). Nine hundred five individuals (49.94% male; age: *M* = 38.22 years, *SD* = 11.64) participated in exchange for $1.60. All respondents provided informed consent.

#### Procedures

Each participant was randomly assigned to see and read about a target that was described as either high or low in honesty-humility and agreeableness. We employed a stimulus-sampling approach to target appearance, which allows for inferences across populations of stimuli as well as populations of participants (Westfall et al., 2014). Each participant was also randomly assigned to see one of 40 different faces (20 male, 20 female) selected from the Chicago Face Database (Ma, Correll, & Wittenbrink, 2015). Given arguments that physical attractiveness is treated as indicative of infection risk (Park, van Leeuwen, & Stephen, 2012), we aimed to sample from a range of attractiveness levels. We identified the man rated most attractive in the normed data set (a rating of 5 on a 1- to 7-point scale based on data reported by Ma et al.) and selected him and 19 other men, each with an attractiveness rating 0.15 scale units below the previous face. We then selected female targets that matched the male targets on attractiveness ratings.

In both conditions, participants read a description of the target. This description was based on items from each of the four facets of honesty-humility (sincerity, fairness, greed avoidance, and modesty) and each of the four facets of agreeableness (forgiveness, gentleness, flexibility, patience). Low-value targets were described on the low end of each facet, and high-value targets were described on the high end of each facet (for complete descriptions, see the Supplemental Material). After seeing the target and reading the description, participants completed the same contact-comfort items used in Studies 1 and 2 (α = .91) and the same 30-item, three-anchor WTR measure used in Study 2 (α = .97). They also rated the target’s honesty and kindness (on 11-point scales) as well as other characteristics (for a complete list, see the Supplemental Material). As intended, relative to targets high in prosocial personality traits, targets low in prosocial personality traits were rated less honest (*M* = 9.53 vs. *M* = 2.39, *p* < .001) and less kind (*M* = 9.33 vs. *M* = 2.93, *p* < .001). Further details are provided in the Supplemental Material.

#### Data exclusion

We excluded 40 participants whose descriptions of the target were nonsensical or demonstrated poor English, eight participants who described their gender identity as neither male nor female, and 30 participants with more than two switch points within any of the three WTR anchors. Results reported below are based on the remaining 827 participants. All outcomes of null-hypothesis significance testing (i.e., *p* < .05) remained when no exclusions were made.

### Results

Using a random-effects model, we regressed contact comfort on target condition (high value vs. low value) and initially modeled random intercepts for stimuli and random slopes for the effect of interpersonal value across stimuli. We removed random slopes that prevented model convergence. People were more comfortable with exposure to pathogens when the targets were described as high in prosocial personality traits (*M* = −0.93, 95% CI = [−1.04, −0.81]) than when they were described as low in prosocial personality traits (*M* = −1.8, 95% CI = [−1.89, −1.70], *F*(1, 822) = 126.1, *p* < .001. When WTR was added as a predictor, both WTR and the manipulation were related to comfort with exposure to pathogens (both *p*s < .001; see Fig. 2). The effect of the manipulation and WTR remained when we further controlled for participant pathogen-disgust sensitivity, participant sex, and target sex, *p*s < .001. Full details are provided in the Supplemental Material.

## Discussion

Results from each of three studies revealed that motivations to avoid infection-risky contact varied markedly across targets with no clear symptoms of illness. Much of this variation was accounted for by targets’ interpersonal value to perceivers. Participants were less averse to infection-risky contact with targets from categories that are, on average, more highly valued (e.g., close friends vs. disliked others), and they were less averse to infection-risky contact with agreeable and honest strangers than with disagreeable and dishonest ones. Further, even within target categories, comfort with infection-risky contact related to a continuous measure of interpersonal value—for example, people who valued their closest friend more were also less averse to infection-risky contact with that friend. We discuss how these findings can inform both the burgeoning behavioral-immune-system literature and our understanding of how infectious disease spreads.

### Implications for understanding the behavioral immune system

The behavioral-immune-system literature largely focuses on understanding how people detect and respond to features that putatively provide information regarding infectiousness, such as pustules and swellings (Ackerman et al., 2018; Murray & Schaller, 2016; Neuberg et al., 2011; Oaten et al., 2011). The current study is a step forward in understanding pathogen avoidance even in the absence of such cues, and it raises critical issues for future research.

First, growing evidence suggests that the behavioral immune system does not output the same pathogen-avoidance motivations across all contexts. It is instead flexible, weighing strands of information to determine the fitness value of contacting another person or item (Neuberg et al., 2011; Tybur & Lieberman, 2016). The current findings demonstrate that interpersonal value is one such strand. Other findings suggest that pathogen avoidance is relaxed in situations that require some exposure to pathogens, such as sexual interactions (e.g., Fleischman et al., 2015) and childrearing (e.g., Case et al., 2006). Future work can test whether relaxed pathogen avoidance toward offspring and mates results only from their high interpersonal value or whether sexual value and genetic relatedness, which inform interpersonal value (but are not redundant with it), additionally shape pathogen avoidance (cf. Tooby et al., 2008).

Second, researchers have speculated that people are more disgusted by infection-risky contact with strangers relative to friends because social familiarity is treated as a cue to infectiousness, just as rashes and sores are (Curtis et al., 2004; Peng et al., 2013; Stevenson & Repacholi, 2005). Similarly, the behavioral-immune-system literature is replete with proposals that prejudices toward members of various groups partially stem from people treating morphological features (e.g., in the cases of the physically disabled, obese, and elderly) or foreign ecological origin (e.g., in the case of immigrants) as cues to infectiousness (for a summary, see Murray & Schaller, 2016). The current results suggest an alternative—or, at least, supplementary—approach to understanding how the behavioral immune system contributes to social biases: Prejudices toward the aforementioned groups might result from perceptions of interpersonal value rather than perceptions of infectiousness. Recent studies has reevaluated claims that anti-immigrant prejudices partially result from perceptions that foreign ecological origin is indicative of infectiousness (e.g., Karinen, Molho, Kupfer, & Tybur, 2019; van Leeuwen & Petersen, 2018); future work could similarly clarify whether the behavioral immune system outputs prejudices toward the obese, elderly, and physical disabled because they are perceived as infectious or because they are perceived as not offering the interpersonal benefits that offset the infection risks posed by any social interaction.

Third, if infection-risky contact is embraced with interpersonally valued others and avoided with interpersonally devalued ones, then contact rituals (e.g., hugs, handshakes) might be used to signal, regulate, and maintain interpersonal valuation. Refusals to engage in such rituals with a specific target might be interpreted as suggesting that the target is not valued enough to risk infection, is perceived as having some symptom of contagious illness, or both. These considerations might contribute to our understanding of the cultural evolution and maintenance of greeting rituals. They also highlight an important limit on the generalizability of these data, which were collected in the United States. Recent findings suggest that at least some contamination aversion exists across human populations (Apicella, Rozin, Busch, Watson-Jones, & Legare, 2018). Universality does not imply an absence of variation, though. Indeed, some evidence suggests that potentially infectious ritualized contact is less prevalent in areas with more infectious disease (Murray, Fessler, Kerry, White, & Marin, 2017). Any signal value of contact and contact avoidance might similarly vary across regions as a function of ecological parasite stress, as might the degree to which interpersonal value influences motivations to embrace or avoid infection-risky contact. Even within a single nation, the relation between interpersonal value and contact comfort might vary as a function of transient infection threats, such as those posed by COVID-19.

### Implications for the spread of infectious disease

Multiple factors constrain the effectiveness of pharmaceutical interventions in combating pandemics. Hence, outcomes of our battles against microbes will hinge on the effectiveness of nonpharmaceutical interventions (Ferguson et al., 2020). In addition to better hygiene (e.g., handwashing), such interventions might focus on stemming contagion within social networks. Indeed, research during the 2009 H1N1 influenza pandemic indicated that infectious disease spreads within social networks much faster than it spreads across the broader population (Christakis & Fowler, 2010). Closer physical proximity and more frequent social interactions doubtlessly contribute to such spread. The current findings reveal another factor that likely exacerbates within-network contagion: the relaxation of pathogen avoidance toward interpersonally valued targets. This observation might help inform approaches to dampening disease transmission during outbreaks. Whereas people need little encouragement to avoid infectious-risky behaviors with most people, they largely feel comfortable engaging in identical behaviors with targets that they especially value interpersonally. Improving our understanding of this and other features of the behavioral immune system can enable a better defense in our war against infectious disease.

## Supplemental Material

Tybur_OpenPracticesDisclosure_rev – Supplemental material for Behavioral Immune Trade-Offs: Interpersonal Value Relaxes Social Pathogen Avoidance Click here for additional data file. Supplemental material, Tybur_OpenPracticesDisclosure_rev for Behavioral Immune Trade-Offs: Interpersonal Value Relaxes Social Pathogen Avoidance by Joshua M. Tybur, Debra Lieberman, Lei Fan, Tom Kupfer and Reinout E. de Vries in Psychological Science

Tybur_Supplemental_Material_rev – Supplemental material for Behavioral Immune Trade-Offs: Interpersonal Value Relaxes Social Pathogen Avoidance Click here for additional data file. Supplemental material, Tybur_Supplemental_Material_rev for Behavioral Immune Trade-Offs: Interpersonal Value Relaxes Social Pathogen Avoidance by Joshua M. Tybur, Debra Lieberman, Lei Fan, Tom Kupfer and Reinout E. de Vries in Psychological Science

## Transparency

*Action Editor:* Eddie Harmon-Jones

*Editor:* Patricia J. Bauer

*Author Contributions*

J. M. Tybur developed the study concept, coordinated data collection, and drafted the manuscript. J. M. Tybur designed Study 1; J. M. Tybur and T. R. Kupfer designed Study 2; and J. M. Tybur, T. R. Kupfer, D. Lieberman, and R. E. de Vries designed Study 3. L. Fan analyzed the data under the supervision of J. M. Tybur. All authors provided critical revisions on the initial draft of the manuscript. All authors approved the final version of the manuscript for submission.

**Open Practices:** All data, analysis scripts, and materials have been made publicly available via OSF and can be accessed at https://osf.io/4agk8/. The design and analysis plans for all three studies were preregistered at https://osf.io/4agk8/. The complete Open Practices Disclosure for this article can be found at http://journals.sagepub.com/doi/suppl/10.1177/0956797620960011. This article has received the badges for Open Data, Open Materials, and Preregistration. More information about the Open Practices badges can be found at http://www.psychologicalscience.org/publications/badges.

## Notes

1. In each study, the main effect of target sex on contact comfort was qualified by an interaction with participant sex. Further details are provided in the Supplemental Material.
