# Supplementary material for: Behavioral Immune Trade-Offs: Interpersonal Value Relaxes Social Pathogen Avoidance
Source: Psychol Sci. 2020 Sep 17;31(10):1211–21. doi: 10.1177/0956797620960011 (PMC7502680; doi:10.1177/0956797620960011)
Supplement: Tybur_Supplemental_Material_rev – Supplemental material for Behavioral Immune Trade-Offs: Interpersonal Value Relaxes Social Pathogen Avoidance [file Tybur_Supplemental_Material_rev.docx]

Behavioral immune trade-offs:

Interpersonal value relaxes social pathogen avoidance

**Supplementary Material**

Figure S1. Welfare-tradeoff ratios across target categories for Studies 1-3


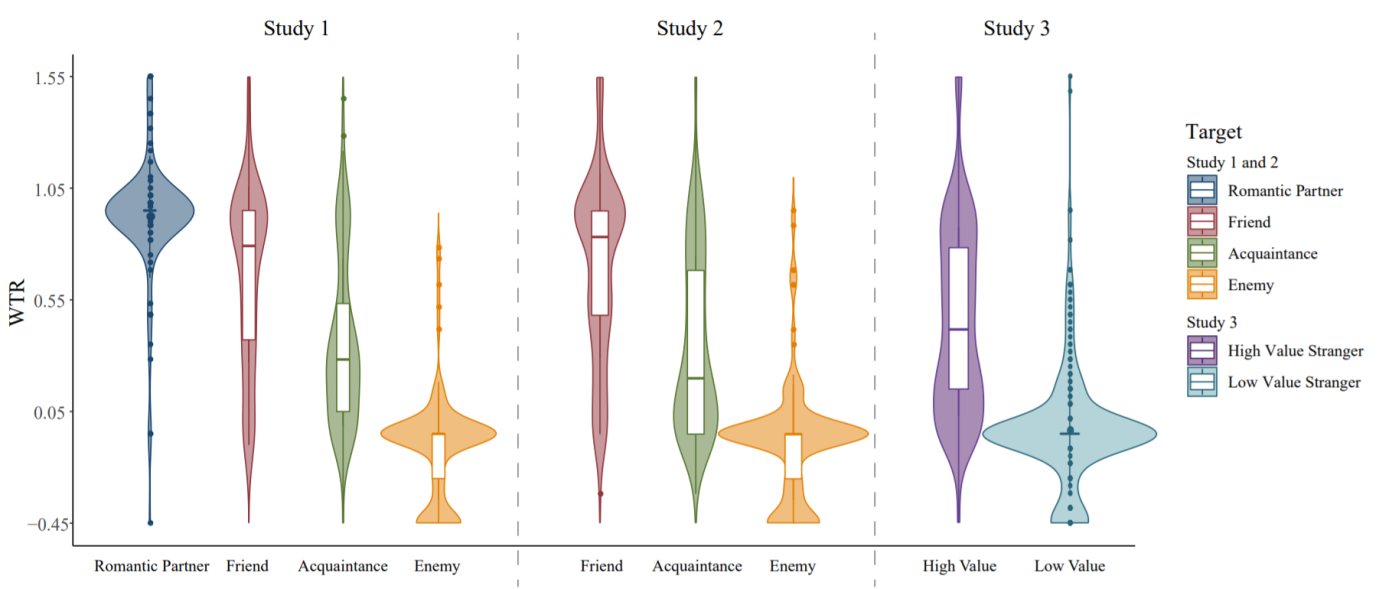


Figure S2. Target ratings for Honesty-Humility for Study 1


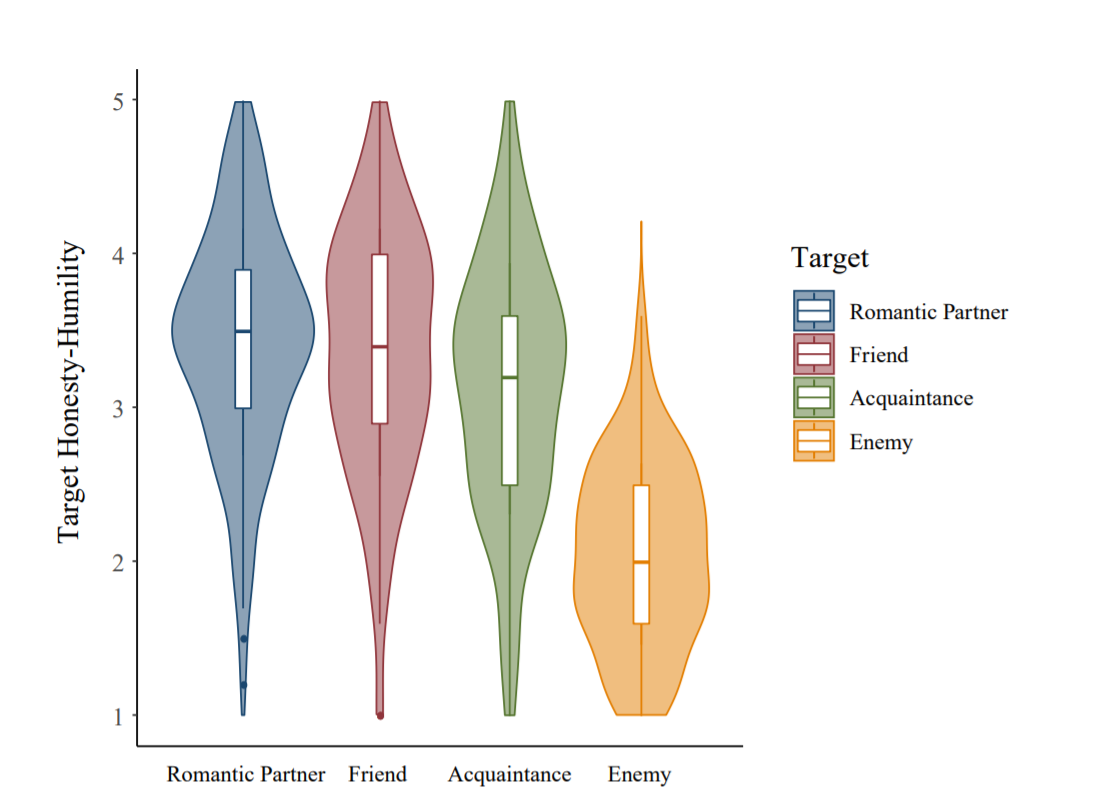


Figure S3. Relations between participant sex and target sex for Studies 1-3.


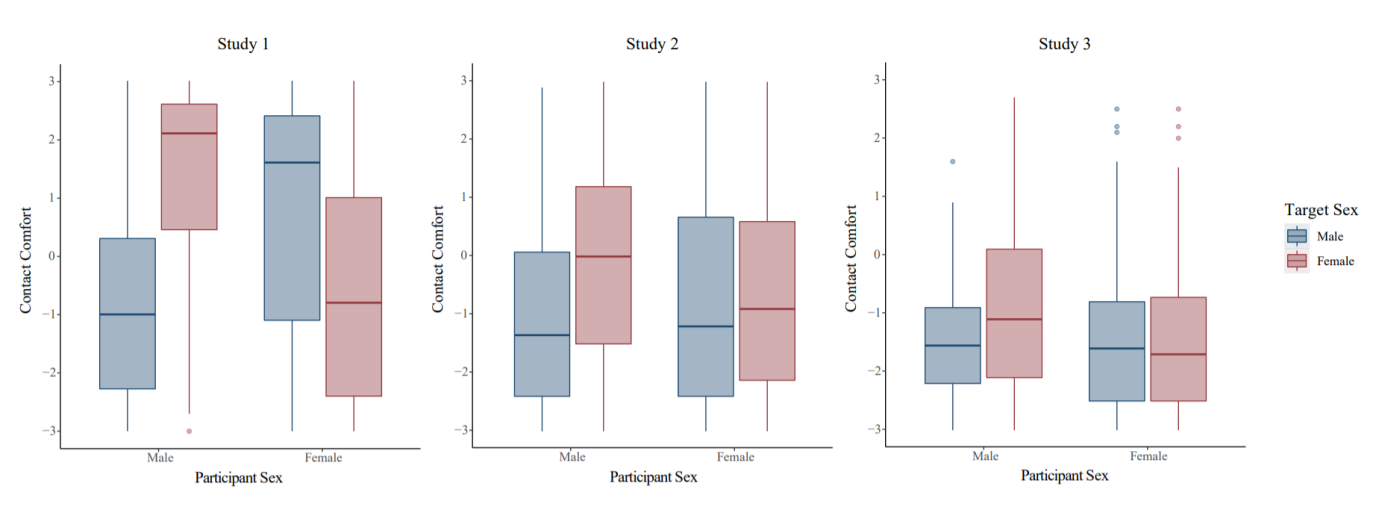
 Interactions between participant sex and target sex in Studies 1-3. The larger effects of target sex in Study 1 were a biproduct of the romantic partner condition; contact comfort was highest for romantic partners, and most romantic partners were opposite sex. We tested the interaction between participant sex and target sex controlling for the variables described in the main manuscript. For Study 1, the interaction was non-zero when added to the third step of the regression model described in the manuscript, *p* = .035. Men were more comfortable with potentially infection contact with female targets than with male targets, *t*(451) = 3.04, *p* = 0.003, but no target sex effect was observed for women, *t*(451) = 0.321, *p* = .748. The same pattern emerged in Study 2, when the same interaction was added to the last step described in the manuscript, *p* < .001; men were more comfortable with potentially infectious contact with female targets than with male targets, *t*(388) = 5.37 < .001, whereas women were equally comfortable with potentially infectious contact with male and female targets, *t*(388) = 0.21, *p* = 0.832. The interaction replicated in Study 3, where targets were strangers, *p* < .001: men were again more comfortable with potentially infectious contact with female targets than with male targets *t*(105) = 4.83, *p* < .001, and women did not discriminate based on target sex, *t*(95) = 1.11, *p* = .272.

Table S1. Study 1 relations between contact comfort, participant variables, and target variables

|  | 1 | 2 | 3 | 4 | 5 | 6 | 7 | 8 |
| --- | --- | --- | --- | --- | --- | --- | --- | --- |
| 1 Contact Comfort | *.96* | **.23** | **-.29** | **.12** | .05 | -.07 | **-.13** | **-.09** |
| 2 WTR | **.68** | *.99* | .08 | **.27** | .03 | **.10** | -.03 | <.01 |
| 3 Disgust Sensitivity | **-.22** | -.09 | *.83* | **-.08** | **-.22** | .01 | .04 | .05 |
| 4 Target Honesty-Humility | **.47** | **.58** | **-.10** | *.88* | -.04 | .04 | **-.12** | .03 |
| 5 Participant Sex | -.02 | .01 | **-.23** | -.01 | *N/A* | **-.10** | -.06 | **-.13** |
| 6 Participant Age | -.07 | .07 | <.01 | .04 | **-.11** | *N/A* | -.04 | **.62** |
| 7 Target Sex | **-.17** | **-.10** | .07 | **-.16** | -.08 | -.06 | *N/A* | -.05 |
| 8 Target Age | **-.19** | **-.10** | -.01 | -.07 | **-.14** | **.61** | .01 | *N/A* |

Bivariate correlations are below the diagonal, coefficient alpha is on the diagonal in italics, and partial correlations controlling for target category are above the diagonal. For target and participant sex, male was coded as 1 and female as 2. Hence, men were lower in disgust sensitivity, but the sexes did not differ in contact comfort. Male targets were rated lower on honesty-humility and evoked lower contact comfort. Bold font denotes *p* < .05.

Table S2. Study 2 relations between contact comfort, participant characteristics, and target characteristics

|  | 1 | 2 | 3 | 4 | 5 | 6 | 7 | 8 | 9 |
| --- | --- | --- | --- | --- | --- | --- | --- | --- | --- |
| 1 Contact Comfort | *.95* | **.34** | **-.30** | -.09 | -.03 | .02 | .05 | **.22** | **-.13** |
| 2 WTR | **.61** | *.97* | **-.19** | .05 | .05 | .08 | -.03 | -.08 | -.02 |
| 3 Disgust Sensitivity | **-.24** | **-.13** | *.82* | -.04 | -.04 | **-.14** | -.05 | .04 | .05 |
| 4 Self Honesty-Humility | -.09 | .04 | -.02 | *.81* | **.38** | **-.09** | **.27** | .04 | **.19** |
| 5 Self Agreeableness | -.06 | -.02 | -.03 | **.37** | *.84* | .04 | .07 | .02 | .02 |
| 6 Participant Sex | .08 | **.12** | **-.15** | -.11 | .03 | *N/A* | -.07 | .01 | **.12** |
| 7 Participant Age | -.08 | -.06 | -.01 | **.26** | .09 | -.09 | *N/A* | .04 | **.58** |
| 8 Target Sex | **-.18** | -.07 | .06 | .02 | .04 | .05 | .03 | *N/A* | **.10** |
| 9 Target Age | **-.21** | **-.15** | .01 | **.19** | .06 | **.21** | **.58** | **.11** | *N/A* |

Bivariate correlations are below the diagonal, coefficient alpha is on the diagonal, and partial correlations controlling for target category are above the diagonal. For target and participant sex, male was coded as 1 and female as 2. Hence, men were lower in disgust sensitivity, but the sexes did not differ in contact comfort. Male targets evoked lower contact comfort. Bold font denotes *p* < .05.

Table S3. Study 3 relations between contact comfort, participant characteristics, and target characteristics

|  | 1 | 2 | 3 | 4 | 5 | 6 | 7 |
| --- | --- | --- | --- | --- | --- | --- | --- |
| 1 Contact Comfort | *.91* | **.20** | **-.36** | **.16** | **.09** | **-.07** | **.14** |
| 2 WTR | **.36** | *.97* | **-.07** | -.03 | -.03 | -.03 | .03 |
| 3 Disgust Sensitivity | **-.33** | -.06 | *.81* | **-.19** | .04 | -.03 | .03 |
| 4 Participant Sex | **.14** | -.03 | **-.20** | *N/A* | **.14** | -.03 | -.04 |
| 5 Participant Age | **-.10** | -.02 | -.05 | **-.15** | *N/A* | .02 | .05 |
| 6 Target Sex | **-.07** | -.01 | -.01 | .05 | .03 | *N/A* | -.03 |
| 7 Target Attractiveness | **.13** | .01 | .02 | .05 | -.06 | .07 | *N/A* |

Bivariate correlations are below the diagonal, coefficient alpha is on the diagonal, and partial correlations controlling for target category are above the diagonal. For target and participant sex, male was coded as 1 and female as 2. Hence, men were lower in disgust sensitivity and higher in contact comfort. Male targets evoked lower contact comfort. Bold font denotes *p* < .05.

Study 3 Manipulation Checks.

Participants rated the targets on height, weight, age, wealth, intelligence, physical attractiveness, kindness, and honesty, and they rated how much they care about Alex. Means differed for all assessments, except for age, *t*(38) = 0.02, *p* = .981, and weight, *t*(38) = 1.57, *p* = .125. The mean difference was small for height, *M*_diff_ = 0.31, *t*(38) = 2.07, *p* = .046, where height was measured on a 20-point scale ranging from “Below 5’0” to “Above 6’6.”

Mean differences were much larger for honesty and kindness (*M*_diff_ = 6.94 and 7.15, respectively) and how much the participant cared about the target (*M*_diff_ = 4.65) than for wealth, intelligence, and physical attractiveness (*M*_diff_ = 0.38, 1.99, and 1.59, respectively). Each of these variables was measured on 11-point scales anchored by “Much less _______ than average” and “Much more ________ than average.”

Study 3 Random Effects Model Details

We intended to model both random intercepts of target faces and random slopes of the predictors in the regression model across target faces. Models including random slopes failed to converge, as did models including target attractiveness. Statistics from the random effects model are presented below. “Value” refers to the manipulation (with low value = 1 and high value = 2), WTRTOTAL refers to welfare-tradeoff ratio, DS refers to pathogen disgust sensitivity, sex refers to participant sex (1 = male, 2 = female), and part_sex refers to target sex (1 = male, 2 = female).


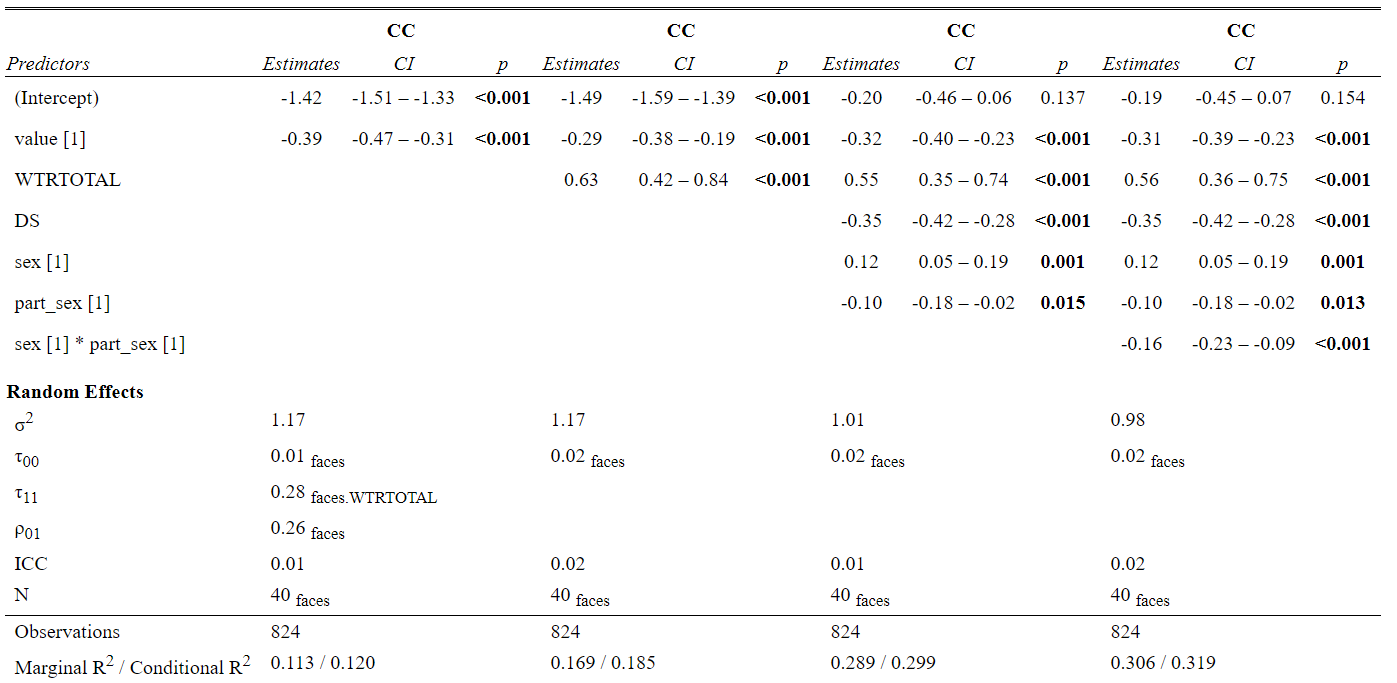


**Contact comfort items**

We generated 10 items inspired by the germ aversion subscale of the Perceived Vulnerability to Disease scale (Duncan et al., 2009), which includes items like “I am comfortable sharing a water bottle with a friend,” and “I dislike wearing used clothes because you do not know what the last person who wore it was like.” The target’s initials were included in both the instructions and the individual items. Participants were given the following instructions:

*Now we would like to know how comfortable you would be with 10 situations involving you and [TARGET]. Please carefully imagine [TARGET] in these situations*.

The 10 items were:

Drinking from the same water bottle as [TARGET].

Using [TARGET]’s deodorant stick on yourself.

Combing your hair with a comb that [TARGET] used earlier.

Touching a handkerchief that [TARGET] had used to blow his or her nose.

Using the same towel that [TARGET] had used to dry him or herself with earlier.

Sitting next to [TARGET] on a bus while he or she was couching and sneezing from a head cold.

Wearing a hat that [TARGET] has worn many times.

Handling [TARGET]’s socks after he or she had worn them while exercising.

Taking a bite out of a sandwich that [TARGET] had been eating.

Putting [TARGET]’s earbuds (in-ear headphones) into your own ears to listen to music.

Each item was rated on a seven-point scale ranging from -3 to +3, with negative and positive anchors labeled “Very uncomfortable” and “Very comfortable,” respectively, and the midpoint labeled “neutral.”

**Welfare-Tradeoff Ratio items**

For Study 1, we used the 60 welfare-tradeoff ratio items described by Kirkpatrick et al., 2015. Based on these 60 items, we estimated switch points for each of six anchor points. Given the high consistency in switch points across anchor points, we used only the $75, $19, and $46 anchor points for Studies 2 and 3. Participants were given the following instructions in each study:

OK, thanks! Next, you will make 30 decisions involving money. For each decision, you will choose whether you get some money, or [TARGET] gets some money. Each decision involves a different amount of money, both for you and for [TARGET]. Here's an example:

Option 1: You get $100, [TARGET] gets $0

Option 2: You get $0, [TARGET] gets $150

If you select option 1, then you would receive $100, but [TARGET] would receive nothing. If you select option 2, then you would receive nothing, and [TARGET] would receive $150.

Please make each decision carefully, and please keep in mind that your decisions involve money for you and for [TARGET]. Your responses are important to us, and we appreciate your efforts!

The table below describes the 60 items used in Study 1. For each item, participants reported their preference between option 1 and option 2, where option 2 corresponded with the target receiving one of the bolded column values and the participant receiving nothing, and option 1 corresponding with the target receiving nothing and the participant receiving one of the non-bolded values in the same column. Participants saw all 60 items in random order in Study 1, and all 30 items from the $75, $19, and $46 columns in random order in Study 2.

| **Ratio** | **Anchor** | | | | | |
| --- | --- | --- | --- | --- | --- | --- |
|  | **$75** | **$19** | **$46** | **$37** | **$23** | **$68** |
| **1.45** | $109 | $28 | $67 | $54 | $33 | $99 |
| **1.25** | $94 | $24 | $58 | $46 | $29 | $85 |
| **1.05** | $79 | $20 | $48 | $39 | $24 | $71 |
| **0.85** | $64 | $16 | $39 | $31 | $20 | $58 |
| **0.65** | $49 | $12 | $30 | $24 | $15 | $44 |
| **0.45** | $34 | $9 | $21 | $17 | $10 | $31 |
| **0.25** | $19 | $5 | $12 | $9 | $6 | $17 |
| **0.05** | $4 | $1 | $2 | $2 | $1 | $3 |
| **-0.15** | $-11 | $-3 | $-7 | $-6 | $-3 | $-10 |
| **-0.35** | $-26 | $-7 | $-16 | $-13 | $-8 | $-24 |

To estimate welfare-tradeoff ratios, we calculated a switch point within each anchor. The switch point refers to the ratio for which participants stop selecting the benefit for themselves and start selecting the benefit for the target. For example, if, for $75 anchor point, the participant preferred to receive $109, $94, and $79, and $64, but preferred to not receive $49, $34, $19, $4, -$11, and $-26, they would have a welfare-tradeoff ratio between .65 and .85 for that anchor point. Hence, we estimate the ratio as .75. If the participant had two switch points within an anchor (e.g., choosing to receive $109 and $94, declining to receive $79, choosing to receive $64, and declining to receive all lower amounts), we took the average of the two switch points (here, the mean of 1.15 and .75, which is equal to .95). If the participant had more than two switch points for any of the six anchors, they were excluded from analyses.

Despite not seeing the ratio values, and despite seeing the items in random order, participants were highly consistent in their switch points across anchors. Indeed, using the procedure described above, alpha coefficients were equal to .99 for Study 1 and .97 for both Studies 2 and 3 (where 30 items for three anchor points were used).

**Note that we departed our Study 1 pre-registered treatment of welfare-tradeoff ratio in two ways. First, we pre-registered estimating WTR for each anchor as the *highest possible* switch point. We instead took the average of switch points if there were multiple switch points. We also pre-registered excluding participants based on the variance in their WTR’s across anchor points. Instead, we excluded participants with more than one switch point across anchor points.**

This departure only applied to Study 1. We used the same procedures to assess WTR in Studies 1-3 (with those used in Studies 2 and 3 following the pre-registered plan).

**Target descriptions (Study 3)**

In the high value (high honesty-humility, high agreeableness) condition, male targets were described as follows (with female target described with female-gendered pronouns):

*This is Alex. People who know him well describe him as flexible and able to get along well with even those he disagrees with. He’s the type of person who wouldn’t cheat or steal from others, even if he could get away with it, and he prefers to be sincere with people rather than manipulating them. Although he earns a good salary, he is not motivated to become extravagantly rich or show off his wealth to others. Indeed, he doesn’t like the idea of people treating him as if he’s superior to them. He is a trusting person who is patient with and non-judgmental of others, and he gives generously to those in need. Ultimately, his coworkers think of him as a kind and honest person who cares deeply for others.*

In the low value (low honesty-humility, low agreeableness) condition, male targets were described as follows (with female targets described with female-gendered pronouns):

*This is Alex. People who know him well complain that he is stubborn and always wants to have things his own way. He’s the type of person who would steal large sums of money if he knew he could get away with it, and he has no problem manipulating people to get what he desires. Although he earns a good salary, he fantasizes about becoming extravagantly rich and showing his wealth off to others. Indeed, he likes the idea of people treating him as if he’s superior to them. He is a bitter person who is impatient with and judgmental of others, and he has little interest in giving to those in need. Ultimately, his coworkers think of him as a dishonest and mean bully who cares primarily about himself.*

**All Measures Collected: Study 1**

A copy of the Qualtrics file used for this study is posted on the Open Science Framework.

Participants completed the following measures, in this order:

1. They reported which of three options best describes their gender (Man, Woman, or Other)
2. They reported their age, in years (open response).
3. They reported whether they are in a romantic relationship (yes or no).
4. They described their annual household income (from <$10,000 to >$100,000 in $5,000 increments).
5. They described themselves on social political issues on a seven-point scale ranging from “very liberal” to “very conservative.”
6. They described themselves on economic political issues on a seven-point scale ranging from “very liberal” to “very conservative.”
7. They reported their generalized social trust on an 11-point scale ranging from 0 (You can’t be too careful) to 10 (Most people can be trusted).
8. They completed the seven-item pathogen disgust subscale of the Three-Domain Disgust Scale (Tybur et al., 2009).
9. They were asked to think carefully about the target (romantic partner, closest friend, acquaintance, or someone they dislike) and record his or her initials.
10. They were asked to describe the target’s physical appearance in a few sentences.
11. They were asked whether the target is a man or a woman.
12. There were asked to report the target’s age (in years).
13. They rated the target on the 10 Honesty-Humility items of the HEXACO-60.
14. They completed the 10 contact comfort items described above.
15. They completed the 60-item welfare-tradeoff ratio task described above.

**All Measures Collected: Study 2**

A copy of the Qualtrics file used for this study is posted on the Open Science Framework.

Participants completed the following measures, in this order:

1. They reported which of three options best describes their gender (Man, Woman, or Other)
2. They reported their age, in years (open response).
3. They reported whether they are in a romantic relationship (yes or no).
4. They described their annual household income (from <$10,000 to >$100,000 in $5,000 increments). They described themselves on social political issues on a seven-point scale ranging from “very liberal” to “very conservative.”
5. They described themselves on economic political issues on a seven-point scale ranging from “very liberal” to “very conservative.”
6. They reported their generalized social trust on an 11-point scale ranging from 0 (You can’t be too careful) to 10 (Most people can be trusted).
7. They completed self-reports of Honesty-Humility and Agreeableness from the HEXACO-60.
8. They completed the seven-item pathogen disgust subscale of the Three-Domain Disgust Scale (Tybur et al., 2009).
9. They were randomly assigned to think of a target (closest friend, acquaintance, someone they dislike) who was either male or female (random assignment crossed with target category) and record his or her initials.
10. They were asked to describe the target’s physical appearance in a few sentences.
11. They were asked how long they’ve known the target.
12. There were asked to report the target’s age (in years).
13. They were asked how many days they’ve been in the same room as the target in the past seven days.
14. They were asked to report the target’s height
15. They were asked to report the target’s weight
16. They were asked how physically attractive people tend to find the target.
17. They were asked how hygienic people tend to find the target.
18. They completed the 10 contact comfort items described above.
19. They completed the 30-item welfare-tradeoff ratio task described above.
20. They briefly described the target’s appearance.
21. They reported how long they’ve known the target.
22. They reported the target’s age, in years (open response).
23. They reported whether they are in a romantic relationship (yes or no).
24. They described their annual household income (from <$10,000 to >$100,000 in $5,000 increments).
25. They described themselves on social political issues on a seven-point scale ranging from “very liberal” to “very conservative.”
26. They described themselves on economic political issues on a seven-point scale ranging from “very liberal” to “very conservative.”
27. They reported their generalized social trust on an 11-point scale ranging from 0 (You can’t be too careful) to 10 (Most people can be trusted).
28. They rated the target on the 10 Honesty-Humility items of the HEXACO-60.
29. They completed the 10 contact comfort items described above.
30. They completed the 30-item welfare-tradeoff ratio task described above.

**All Measures Collected: Study 3**

A copy of the Qualtrics file used for this study is posted on the Open Science Framework.

Participants completed the following measures, in this order:

1. They reported which of three options best describes their gender (Man, Woman, or Other)
2. They reported their age, in years (open response).
3. They reported whether they are in a romantic relationship (yes or no).
4. They described their annual household income (from <$10,000 to >$100,000 in $5,000 increments).
5. They completed the seven-item pathogen disgust subscale of the Three-Domain Disgust Scale (Tybur et al., 2009).
6. They saw one of 40 possible target faces paired with one of two descriptions (high interpersonal value versus low interpersonal value).
7. They estimated the target’s height.
8. They estimated the target’s weight.
9. They estimated the target’s age.
10. They rated the target on wealth.
11. They rated the target on intelligence.
12. They rated the target on physical attractiveness.
13. They rated the target on kindness.
14. They rated the target on honesty.
15. They reported the degree to which they care about the target’s welfare.
16. They completed the 10 contact comfort items described above.
17. They completed the 30-item welfare-tradeoff ratio task described above.
